# TabSQL: a MySQL tool to facilitate mapping user data to public databases

**DOI:** 10.1186/1471-2105-11-342

**Published:** 2010-06-23

**Authors:** Xiao-Qin Xia, Michael McClelland, Yipeng Wang

**Affiliations:** 1 Vaccine Research Institute of San Diego, 10835 Road to the Cure, San Diego, CA 92121, USA; 2 Department of Pathology & Laboratory Medicine, University of California, Irvine, CA 92697, USA

## Abstract

**Background:**

With advances in high-throughput genomics and proteomics, it is challenging for biologists to deal with large data files and to map their data to annotations in public databases.

**Results:**

We developed TabSQL, a MySQL-based application tool, for viewing, filtering and querying data files with large numbers of rows. TabSQL provides functions for downloading and installing table files from public databases including the Gene Ontology database (GO), the Ensembl databases, and genome databases from the UCSC genome bioinformatics site. Any other database that provides tab-delimited flat files can also be imported. The downloaded gene annotation tables can be queried together with users' data in TabSQL using either a graphic interface or command line.

**Conclusions:**

TabSQL allows queries across the user's data and public databases without programming. It is a convenient tool for biologists to annotate and enrich their data.

## Background

In high-throughput genomics and proteomics studies, raw data and the results of analyses are usually organized in a table-like format (e.g., [1-3]), with rows representing genes or probes, while columns denote experimental features and annotations, such as sample identifiers, gene annotations, fold changes in signal, or the *p *values of statistical analyses. It is common for biologists to want to add additional annotations. There are public databases that can be useful sources of such annotations. The Gene Ontology (GO) project http://www.geneontology.org develops and maintains a controlled vocabulary of gene and gene product attributes, and provides detailed and unified gene and gene products annotation files [4,5]; the Ensembl project http://www.ensembl.org offers genome databases for a variety of organisms [6]; the UCSC genome bioinformatics site http://genome.ucsc.edu/ has also been widely used by researchers from all over the world [7].

Microsoft Access http://office.microsoft.com/access is a commonly used desktop database management system that biologists use to view, map and query large data files. However, Access does not provide any tools to directly link to public databases in genome research. Thus, we designed TabSQL, a generic tool for inquiry within or across table-like data files in which we have implemented preset functions to download and install data files from useful databases.

## Implementation

### Prerequisites

MySQL http://www.mysql.com is the search engine in TabSQL, therefore the user needs to have a user account on a MySQL server. The user can access a dedicated MySQL server or, more likely, install MySQL on their local computers. Upon first running TabSQL, the user will be asked for the information regarding the MySQL account/sever, or the MySQL administrator account to create a new user account. TabSQL is written in pure Python. It needs Python 2.3 or later versions with three packages, which are not included in the Python standard library. These three additional packages are: wxPython http://www.wxpython.org for the graphic interface development; pycrypto http://www.pycrypto.org/ for encryption; and MySQLdb http://mysql-python.sourceforge.net/ for the interface between TabSQL and the MySQL server. TabSQL can run on a variety of operating systems (OS), including Microsoft Windows and many POSIX systems. It has been tested on Windows 2000, Windows XP, Windows 7, and a series of GNU/Linux systems, including CentOS 4.X and 5.X, Fedora Core 3, Fedora Core 6, Fedora 9, Fedora 12, and Ubuntu 8.04 to 9.10. TabSQL should work on other OS environments if the three additional Python packages are installed. A TabSQL tutorial with some detailed application examples is available in the help document online http://www.webarray.org/softwares/tabsql/help.html.

### Interface

**Figure 1:**
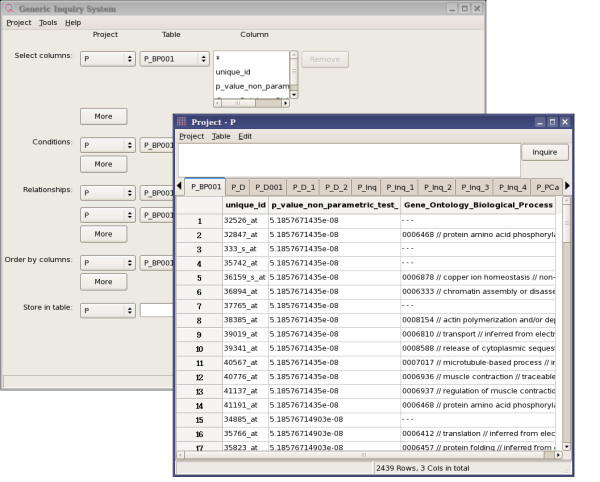
**The interface of TabSQL**. The main window and a project window are shown. More than one project window can be opened at the same time.

The graphic interface of TabSQL consists of a main window and one or more project windows (see Figure 1).

#### Main window

The main window is a graphic interface for querying tables for subsets of data which will subsequently be shown in project windows. From the main window, we can also create/open/delete projects.

#### Project window

Each opened project will be displayed in a project window, which shows data included in the project in spreadsheets. Results from queries will be added to a project and shown in the project window.

### Data organization

TabSQL allows users to cross-reference their data with imported databases and then filter using their own queries. The MySQL open source database management system http://www.mysql.com is adopted as the database engine. All data in TabSQL are stored in a MySQL database, where data are organized as projects. A project consists of a series of database tables that share the project name as a prefix on all table names. Data can be imported into a project after it has been created or opened. TabSQL can use a MySQL server on the local computer or on another computer on the same intranet. Although one can use a MySQL server through the internet, this is not desirable because of the slow transfer rate for bulk data and security issues such as SQL injection attacks. If the user needs to use an internet connection to access a MySQL server, we suggest the use of a SSH (Secure Shell) tunnel to forward the remote MySQL port to the local computer, thereby encrypting communications between TabSQL and the MySQL server.

### Data sources

TabSQL aims to help users to annotate their data with annotations from public databases.

#### User data

Typically, user data are organized in a table-like format, displaying column names in the header row. These data should be saved in TAB-delimited ASCII files, which can be easily imported into a TabSQL project by the "*Add*" command in the menu of the project window. TabSQL automatically determines the data type for each column after scanning the user data, and creates a database table with suitable structure, in which the user data are deposited. If the user data exist in other formats, they should be converted to TAB-delimited ASCII files. Such a conversion is a standard feature of most spreadsheet programs. In some cases the user data are available as a MySQL dump package. The user can load the data into a MySQL database then import it to TabSQL by using the "*Import*" command, which is designed to import tables from databases on the same MySQL server.

#### Public databases

Some public databases provide MySQL format data files or TAB-delimited ASCII text files for download. Data in such formats can be imported to TabSQL in the same way as user data. TabSQL provides menu commands for automatic downloading and depositing for subsets of three well-known genome annotation public databases - the GO database, the Ensembl databases, and the UCSC genome databases. GO provides three subsets for download - "termdb", "assocdb", and "seqdb", which are presented to the user as options. Because the Ensembl databases and the UCSC genome databases are collections of multiple databases for different organism species, TabSQL will open a window to allow the user to select the database of interest when the user clicks on the menu command. All necessary files will be downloaded from the FTP server of the public database and saved into a temporary directory on the local computer, subsequently all tables will be loaded into the current TabSQL project. For frequently updated public databases such as GO, the user can synchronize the local copies with the latest data available online by reusing the same command as was used to download the data in the first instance. TabSQL will alert the user if an imported public database has not been updated in a given time span.

The speed of the internet connection can be a bottleneck for the introduction of public databases into TabSQL. The time cost for this introduction is mostly determined by the download speed. Our test on a client in a local network with a T1 internet connection (1.544 mega bits per second), determined that the introduction or updating of the GO "termdb" database can be done within one minute. The sizes of databases on Ensembl or UCSC bioinformatics site vary drastically, and so do download times. Downloads take from minutes to hours depending on which subset is chosen by the user.

Although TabSQL is designed to query with the specified public databases, these databases are not mandatory components of TabSQL. TabSQL can be used solely to manipulate and query users' data files. In addition, automatic access to additional popular databases will be added to TabSQL by the authors upon user request.

### Making queries

There are two approaches to make queries, either by using the Graphic User Interface (GUI) in the main window or by typing commands into the Command Line Interface (CLI) in project windows. There are examples with detailed guides in the online document for both approaches. Each query will generate a new table in a project. Users can "Copy", "Paste" or "Save" the contents of a table. TabSQL communicates with the MySQL server using Structured Query Language (SQL). SQL statements used for queries can be saved as a text file, which can be used later to help beginners to learn SQL syntax.

Using GUI, queries can be made by clicking on options in the main window. There are five sections for making queries. These sections are used to determine columns to be selected for output, to specify searching criteria, and to sort and name result tables. Based on chosen options, TabSQL automatically constructs an SQL "SELECT" command and submits it to the MySQL server. Considering the fact that query performance can be significantly improved on indexed tables, a menu command in the project window is provided to build indices on tables.

The GUI is very easy to use even for beginners. However, the Command Line Interface (CLI) presents a more flexible way to construct queries. By typing commands in the project window, the user can make very sophisticated and complicated queries. TabSQL supports complete syntax of three SQL commands - "SELECT", "ALTER", and "UPDATE". These commands are used for making queries, changing the structures/contents of tables, or creating indices on tables. When operating on a single table, TabSQL allows commands in three simplified forms, in which only part of the complete SQL statement need to be typed.

• The "SET" clause from an "UPDATE" command. For example, the command "set columnA = 5 where columnB > 10" will change the value in columnA to 5 if columnB has a value greater than 10 at the same row.

• The "ORDER BY" clause from a "SELECT" command. To sort the active table by a column "score", the user can simply type "order by score" for ascending order, or "order by score desc" for descending order.

• The "WHERE" clause from a "SELECT" command. To use the "WHERE" clause solely, the keyword "WHERE" should not be typed, e.g. a command can be simple as "*p *< 0.01" or "*p *< 0.01 order by *p*". The former allows the data to be filtered by keeping the genes at significance level 0.01, and the latter can further sort the genes by the *p *values.

These simplified commands should abide by the syntax of the corresponding clauses because TabSQL directly uses these commands as a part of a complete SQL statement.

## Discussion and Conclusion

TabSQL has three significant features: (1) TabSQL will automatically build a table from a tab-delimited file; (2) TabSQL will build tables from external databases like GO; and (3) TabSQL will run queries through a GUI. Easy import of public databases and loading user data into the database makes TabSQL convenient for integrating these with each other. By querying across tables without programming, biologists are able to annotate and screen their data easily. MySQL-based TabSQL allows a far larger number of records to be imported compared to Microsoft Excel. This feature is especially important when dealing with huge high-throughput genomics data sets. Thus, TabSQL is a useful tool for biologists to annotate and enrich their data.

## Availability

TabSQL is an open source software package distributed under the GNU General Public License http://www.gnu.org/licenses/gpl.txt. The source code is free to download at http://www.webarray.org/softwares/tabsql, or at http://tabsql.sourceforge.net. Compiled executables are also provided for Windows.

## Authors' contributions

All authors participated in the design and testing of the software. XQ coded the software and drafted the manuscript. MM and YW contributed to the final version of the manuscript. All authors read and approved the final version of the manuscript.
